# Magnetic maps in animal navigation

**DOI:** 10.1007/s00359-021-01529-8

**Published:** 2022-01-09

**Authors:** Kenneth J. Lohmann, Kayla M. Goforth, Alayna G. Mackiewicz, Dana S. Lim, Catherine M. F. Lohmann

**Affiliations:** Department of Biology, University of North Carolina, Chapel Hill, NC 27599 USA

**Keywords:** Magnetoreception, Navigation, Natal homing, Philopatry, Migration

## Abstract

In addition to providing animals with a source of directional or ‘compass’ information, Earth’s magnetic field also provides a potential source of positional or ‘map’ information that animals might exploit to assess location. In less than a generation, the idea that animals use Earth’s magnetic field as a kind of map has gone from a contentious hypothesis to a well-established tenet of animal navigation. Diverse animals ranging from lobsters to birds are now known to use magnetic positional information for a variety of purposes, including staying on track along migratory pathways, adjusting food intake at appropriate points in a migration, remaining within a suitable oceanic region, and navigating toward specific goals. Recent findings also indicate that sea turtles, salmon, and at least some birds imprint on the magnetic field of their natal area when young and use this information to facilitate return as adults, a process that may underlie long-distance natal homing (a.k.a. natal philopatry) in many species. Despite recent progress, much remains to be learned about the organization of magnetic maps, how they develop, and how animals use them in navigation.

## Introduction

Life arose in Earth’s magnetic field. Although the geomagnetic field has waxed, waned, and shifted over time, it has been present continuously for as long as organisms have existed. Moreover, the field is present everywhere on and near the planet, from the highest altitude where windborne microbiota are lofted to the deepest trenches of the sea. Given its ubiquity, it is perhaps not surprising that numerous animals have evolved the ability to detect Earth’s magnetic field and use it to guide their movements over a variety of spatial scales.

Animals can potentially derive two different kinds of information from Earth’s magnetic field. Many species have a magnetic compass, meaning that they use the magnetic field as a source of directional information that allows them to set and maintain headings, for example, to the north or south. Some animals, however, use Earth’s magnetic field as a source of positional information which can be used to assess geographic location. Animals with this ability are said to possess a ‘magnetic map’ (Lohmann et al. 2007).

It is important to recognize that the term ‘magnetic map’ is widely used as a convenient, catch-all descriptor encompassing all uses of geomagnetic positional information by animals; thus, the term carries with it no assumptions about the nature of the internal spatial representation, if any, that an animal has (Lohmann et al. 2007; Henshaw et al. 2010; Gould 2014; Putman et al. 2015). The information in a magnetic map can be learned or inherited, specific or very general, and used for a variety of purposes. A magnetic map might, for example, tell an animal that it has reached a point in a migratory route where it should change direction, that it is approaching the boundaries of an oceanic feeding area, that it has returned to an area of origin after a long migration, or that it is approximately north or south of a place where it lives. Thus, in the lexicon of the animal navigation literature, an animal has a magnetic map if it derives positional information from Earth’s magnetic field; it has a magnetic compass if it uses the geomagnetic field to maintain direction. Some animals, of course, have both.

The discovery of the magnetic map sense has revolutionized studies of animal navigation and transformed our understanding of how animals guide themselves, especially over long distances. In little more than 2 decades, the concept of magnetic maps has gone from a speculative and controversial idea to a widely accepted phenomenon. Indeed, magnetic maps now appear likely to explain many of the most impressive navigational feats in the animal kingdom.

In this review, we summarize what is known about magnetic maps in animals. We begin with a description of positional information in Earth’s magnetic field and a brief history of research on magnetic maps. We then summarize evidence for magnetic maps in different animals, highlighting two types of maps: one used by first-time migrants to guide movements along migratory pathways and apparently based largely on inherited information, the other involving navigation to a goal and based on information that is partly or entirely learned. We next discuss magnetic maps in the context of geomagnetic imprinting, a process that may largely explain how sea turtles, salmon, and at least some birds return to their area of origin after long migrations. Finally, we explore how magnetic maps might be organized and highlight promising areas for future research.

## Key concepts

### Positional information in Earth’s magnetic field

**Fig. 1:**
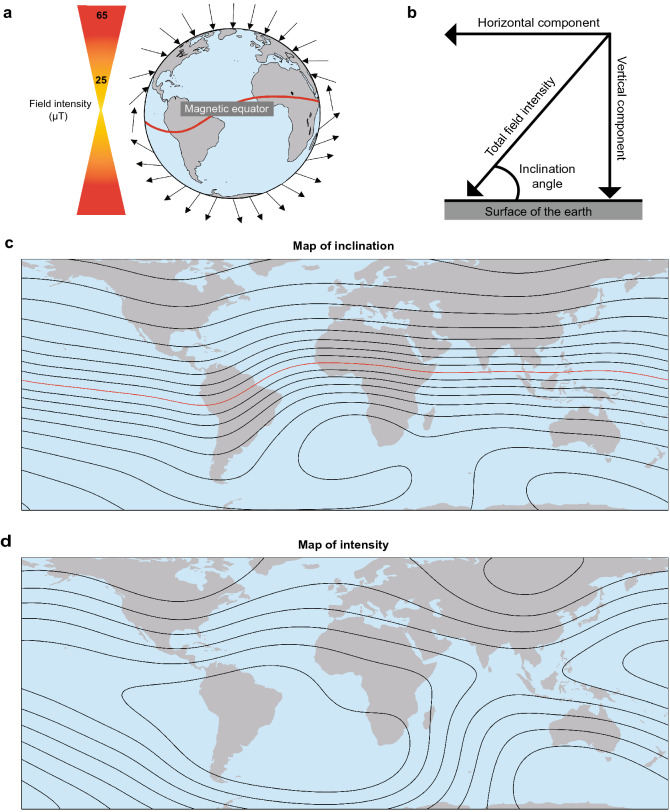
Earth’s magnetic field. **a** Diagram illustrating how field lines (represented by arrows) intersect Earth’s surface, and how inclination angle (the angle at which field lines intersect Earth’s surface) varies with latitude. At the magnetic equator (the curving line across the earth), field lines run parallel to the earth’s surface. Field lines become progressively steeper as one travels north toward the magnetic pole, where the field lines are directed straight down into the earth and the inclination angle is 90°. The intensity (strength) of the field varies in a slightly different direction than inclination; intensity is strongest near the magnetic poles and weakest near the equator. **b** Diagram illustrating four elements of geomagnetic field vectors that might, in principle, provide animals with positional information. The field present at each location on Earth is defined by a total field intensity and an inclination angle. The total intensity can be resolved into two vector components: the horizontal field intensity and the vertical field intensity. (Whether animals can resolve the total field into vector components is not known.) **c** Isolines of inclination are shown in 10° increments. Over much of the globe, inclination is strongly correlated with latitude and is thus potentially useful in a magnetic map. **d** Isolines of total field intensity shown in increments of 5 µT. Maps of magnetic isolines were derived from the World Magnetic Model for 2021 (Chulliat et al. 2020)

In its overall structure, the magnetic field of the earth resembles the dipole field of a bar magnet, with field lines emerging from the southern hemisphere and curving around the planet to re-enter in the northern hemisphere (Fig. 1a). Because of its geometry, the geomagnetic field varies predictably across the globe; thus, animals might hypothetically use several different magnetic parameters to assess their position. For example, at each location, the magnetic field lines intersect Earth’s surface at a specific angle of inclination, with the angle becoming progressively steeper as one moves from the magnetic equator towards the magnetic poles (Fig. 1b, c). Similarly, the total intensity, or field strength, is generally strongest near the magnetic poles and weakest near the magnetic equator, but the exact pattern of variation differs from that of inclination (Fig. 1d). The intensity of the horizontal field and vertical field (Fig. 1b) also vary predictably across Earth’s surface, although whether animals can resolve the magnetic field into its vector components is unknown. Finally, for animals such as birds that can potentially perceive the direction of true geographic north (e.g., using star patterns to determine Earth’s axis of rotation), additional magnetic parameters such as declination (the difference between true north and magnetic north) might also be used.

The overall pattern of the main dipole field is potentially useful for navigation because of its predictability (e.g., Fig. 1c, d). In some locations, however, the global pattern is disrupted by magnetic anomalies caused by concentrations of magnetic minerals in the earth’s crust. Although anomalies are typically small relative to the main dipole field, they are often associated with steep gradients (i.e*.,* variation per distance) of intensity and inclination that can be aligned in directions differing from the overall pattern of the dipole field (Skiles 1985; Johnsen and Lohmann 2005). Since anomalies vary greatly in size, strength, and other characteristics, and because different animals move over vastly different spatial scales, it is difficult to generalize about how anomalies might affect magnetic navigation, although they may function as landmarks for some species (Box 1).

Box 1 Magnetic anomalies Magnetic anomalies are typically caused by geological features in the upper few kilometers of Earth’s crust (Skiles, 1985). Although exceptions exist, most anomalies do not exceed 1% of Earth’s total field (Johnsen and Lohmann 2005; Maus et al. 2009). Given the diversity of both magnetic anomalies (Skiles 1985) and animal movements, it is difficult to generalize about what effects anomalies might have on animal navigation. In some cases, animals that migrate long distances might learn to recognize magnetic anomalies and use them as landmarks (e.g., Jones and MacFadden 1982; Skiles, 1985; Lohmann 1991; Walker et al. 2003). Indeed, some whales have been hypothesized to exploit magnetic anomalies associated with seafloor spreading zones as pathways for migratory movements (Klinowska 1985; Kirschvink et al. 1986; Walker et al. 1992). Several other means have been proposed by which animals might exploit local magnetic contours and gradients for navigation, even over short distances (e.g., Klimley 1993; Walker 1998; Phillips et al. 2006; Dennis et al. 2007).

Interestingly, magnetic anomalies sometimes, though not always, disrupt the homing behavior of pigeons (Walcott 1978; Wiltschko et al. 2010). Results suggest that the location of lofts where pigeons are raised, and the navigational cues available to the birds early in life, may determine whether anomalies affect homing in a given situation (Walcott, 1992). Collectively, these and other findings suggest that magnetic anomalies can facilitate animal navigation, impair it, or have no effect, depending on circumstances and species. Although much remains to be learned, navigational strategies that exploit the magnetic topography of local anomalies appear likely to be site-specific, difficult to generalize, and learned rather than inherited. Our review focuses primarily on the use of magnetic positional information in the main dipole field of the earth (Fig. 1). This information, unlike localized anomalies, can be exploited continuously by animals that migrate long distances.

The strength of local anomalies decreases rapidly with distance from them. Thus, for animals such as migratory birds and sea turtles that typically fly or swim far above geological formations, such anomalies may often be of little consequence, inasmuch as animals moving rapidly through small anomalous regions may experience only slight, transient irregularities before re-entering a magnetic environment dominated by the much larger main (dipole) field. Animals that crawl across the substrate and only move over short distances, however, inhabit a world in which the magnetic environment they experience can be influenced greatly by local anomalies. Thus, the spatial scale over which an animal travels, its speed, and its proximity to the Earth’s surface are all important factors in evaluating the magnetic landscape in which an animal navigates.

**A brief history of magnetic maps in animals**

The idea that animals use Earth’s magnetic field as a kind of map for determining geographic position was first proposed more than a century ago (Viguier 1882). At the time, no credible evidence existed that animals detect magnetic fields and the concept thus gained little traction. Nearly a century later, however, when evidence for magnetic sensitivity in several animals began to emerge, the concept of magnetic maps was again proposed and developed in greater detail, particularly in the context of navigation by homing pigeons (Gould 1980, 1982; Moore 1980; Walcott 1980). At about the same time, findings from several other animals began to hint at the possibility of magnetic maps (Rodda 1984; Beck and Wiltschko 1988; Tesch et al. 1992), but the idea continued to meet with considerable skepticism and resistance through the end of the millennium (Courtillot et al. 1997; Wallraff 1999; Papi 2001).

The first direct evidence that animals use specific parameters of Earth’s magnetic field as a kind of map came from experiments with sea turtle hatchlings (Lohmann and Lohmann 1994, 1996a). These studies investigated whether loggerhead turtles (*Caretta caretta*) from Florida, U.S.A.*,* might use magnetic positional information to help them remain within the North Atlantic Subtropical Gyre, a circular, warm-water current system in which the young turtles spend several years before returning to the North American coast (Carr 1986). In an initial experiment (Lohmann and Lohmann 1994), hatchlings were tethered in a water-filled arena surrounded by a magnetic coil system used to generate earth-strength magnetic fields with different inclinations, while the intensity of the field was held constant. Turtles exposed to a field with an inclination angle found along the northern boundary of the North Atlantic gyre swam south-southwest. In contrast, hatchlings exposed to an inclination angle found near the southern boundary of the gyre swam in a northeasterly direction. These findings revealed that loggerheads distinguish among different inclination angles, and that inclination angles found near the northern and southern gyre boundaries elicited orientation that directs turtles back toward the gyre center. The results were, therefore, consistent with the hypothesis that specific inclination angles in effect warn turtles that they have reached the latitudinal extremes of the gyre and must adjust swimming direction to avoid straying into unfavorable oceanic regions.

In a subsequent experiment, the inclination of the field was held constant while the intensity was varied (Lohmann and Lohmann 1996a). Turtles exposed to a field with an intensity matching one near North Carolina, on the east coast of the U.S.A., swam eastward. A second group exposed to a field with an intensity that the turtles first encounter near Portugal swam westward. Thus, turtles can distinguish among field intensities that exist along their migratory route. Moreover, because both eastern orientation near North Carolina and western orientation near Portugal would presumably function to keep young turtles within the gyre, the results imply that turtles can derive positional information from field intensity. Taken together, these two studies demonstrated for the first time that an animal can detect two magnetic parameters, inclination and intensity, that might function in a magnetic map sense. These two parameters vary in somewhat different directions across much of the globe and thus form a bicoordinate magnetic grid of sorts (Lohmann and Lohmann 1996a, 1996b). Suddenly, the concept of magnetic maps seemed very plausible (Lohmann et al. 1999).

Studies with salamanders soon revealed that turtles are not unique in deriving positional information from magnetic parameters (Fischer et al. 2001). The red-spotted newt (*Notophthalmus viridescens*), when exposed to a magnetic inclination angle that exists 200–400 km north of the home area, oriented southward, while newts exposed to an inclination angle that exists south of the home area oriented northward. Thus, newts, like sea turtles, can detect inclination angle and use it to assess position. An additional experiment revealed that newts can detect changes in inclination of at least 0.5° (Phillips et al. 2002).

**Magnetic displacement experiments: a key technique**

The experiments with turtles and newts established that animals detect magnetic parameters that might function in magnetic maps. In these initial studies, however, either intensity or inclination was held constant while the other was varied. This approach was necessary to demonstrate that animals detect each magnetic parameter. In nature, however, these field elements vary together across Earth’s surface. Thus, most pairings of inclination and intensity used in these early studies do not exist anywhere in the world.

In many subsequent experiments, sea turtles and other animals have been exposed to magnetic fields that replicate those existing at various distant locations, and the orientation responses to these fields have been observed. This technique has come to be known as ‘magnetic displacement’, meaning that animals are not physically moved from the testing site, but are instead exposed to magnetic fields that exist elsewhere (Putman 2018). An advantage of the approach is that all other cues present at the test location, aside from the magnetic field, remain unchanged. Thus, if a change in behavior occurs, it can be attributed to magnetic cues. Magnetic displacement experiments have provided a powerful tool for demonstrating the existence of magnetic maps in diverse animals.

**Functions of magnetic maps**

Evidence has emerged that different animals exploit magnetic positional information in different ways and for different purposes, including facilitating movements along vast migratory pathways, helping animals remain within an appropriate oceanic area, navigating towards a particular goal, and relocating natal areas for reproduction. It is noteworthy that, in some cases such as sea turtles, the magnetic map sense of a single species is used in different migratory and behavioral contexts throughout the animal’s life, depending on what is needed at a particular life history stage. Although no system of categorization fully captures the complexity of magnetic maps, a useful starting point is to highlight two general types: those used by first-time migrants to guide movements along migratory pathways, and those used by animals to navigate toward a specific goal.

**Magnetic maps and migratory pathways**

Several groups of animals that migrate when young are now known to have magnetic maps which help them navigate along migratory pathways and/or remain in appropriate geographic areas (e.g., Lohmann et al. 2001, 2012; Putman et al. 2014c, 2020). In most such cases, magnetic fields that exist in particular geographic regions elicit directional changes at crucial locations and boundaries. These responses appear to be innate, inasmuch as the animals studied had never migrated, but responded to specific fields that exist along the migratory pathway the first time they experienced them. For expedience, maps of this type are sometimes referred to as ‘inherited magnetic maps’ (Putman et al. 2014c), though this term should not be interpreted to mean that the map is exclusively under genetic control. Indeed, magnetic fields present during development and/or early life appear to influence the responses in ways that are not yet understood (e.g., Fuxjager et al. 2014; Putman et al. 2014b), suggesting that early experience may play a role.

**Magnetic maps used in goal navigation**

Some animals have magnetic maps that facilitate navigation to specific target areas, such as locations used in foraging, sheltering, or reproduction (e.g., Boles and Lohmann 2003; Lohmann et al. 2004; Kishkinev et al. 2015). As a general rule, magnetic maps associated with goal navigation are likely to depend largely on learned information, although exceptions may exist (Gould 2014; Putman et al. 2014c; Putman 2021). Natal homing (also known as natal philopatry), in which migratory animals return to an area of origin to reproduce after first migrating a considerable distance away, can be considered a special form of goal navigation.

## Evidence for magnetic maps

Evidence for magnetic maps has been acquired in diverse animals. Here, we summarize what is known about magnetic maps in different animal groups, with emphasis on how magnetic positional information facilitates migratory movements at different life history stages. The special case of geomagnetic imprinting and natal homing is considered separately at the end of the section.

### Sea turtles

Sea turtles are reptiles and must lay their eggs on land. Most species and populations have similar life histories (Bolten 2003). Hatchling turtles emerge from underground nests, scramble across the beach to the sea, and undertake long-distance migrations that, in some populations, span entire ocean basins. Juveniles of most species eventually take up residence in coastal waters. Adults migrate between feeding and nesting areas throughout their lives, with females typically returning to their natal region to nest. As will be discussed, evidence indicates that hatchlings begin their first migration with an inherited magnetic map in which regional magnetic fields serve as open-sea navigational markers and elicit changes in swimming direction at crucial locations. By contrast, older sea turtles use magnetic maps in goal navigation, both to arrive at foraging sites and to return to their natal region.

#### Inherited magnetic maps in hatchling sea turtles

Studies with young sea turtles have focused on hatchling loggerhead turtles from Florida, U.S.A., which migrate offshore to the Gulf Stream, become entrained within the North Atlantic Subtropical Gyre, and gradually circle the north Atlantic Ocean before returning to the North American coast (Carr 1986). In an initial study using magnetic displacements (Lohmann et al. 2001), hatchlings were tested in three magnetic fields that exist at widely separated locations along the migratory route. In response, turtles swam in directions that would, in each case, help them remain within the gyre and advance along the migratory pathway. The results demonstrate that young loggerheads begin their transoceanic migration with a kind of magnetic map in which regional magnetic fields function, in effect, as navigational markers. Given that the turtles were collected directly from nests and had never been in the ocean, the responses appear to be inherited. Natural selection presumably modifies these responses over time as Earth’s field gradually changes (Box 2).

Box 2: inherited magnetic maps and secular variation Earth’s magnetic field changes over time; changes in field elements such as inclination and intensity are referred to as secular variation (Skiles 1985). An interesting question is how turtles, fish, and other animals can evolve behavioral responses to magnetic fields in different geographic areas despite secular variation (Lohmann and Lohmann 2003). A likely answer is that strong selective pressure maintains an appropriate coupling between the responses of animals and the magnetic fields that exist at crucial geographic locations at any point in time (Lohmann et al. 2001, 2012; Putman et al. 2011). For example, young turtles and fish that stray out of thermally appropriate oceanic areas are quickly eliminated from the population, while those with orientation responses that keep them in favorable regions survive to pass on their genes. In this way, strong selective pressure may ensure that the responses of animals evolve rapidly in response to the changing geomagnetic field. It is also possible that the field in which an animal develops influences the ontogeny of the magnetic map (Putman et al. 2014b; Fuxjager et al. 2014), providing a way for animals in each generation to adjust responses (perhaps behaviorally, developmentally and/or epigenetically) relative to changing field conditions.

**Fig. 2:**
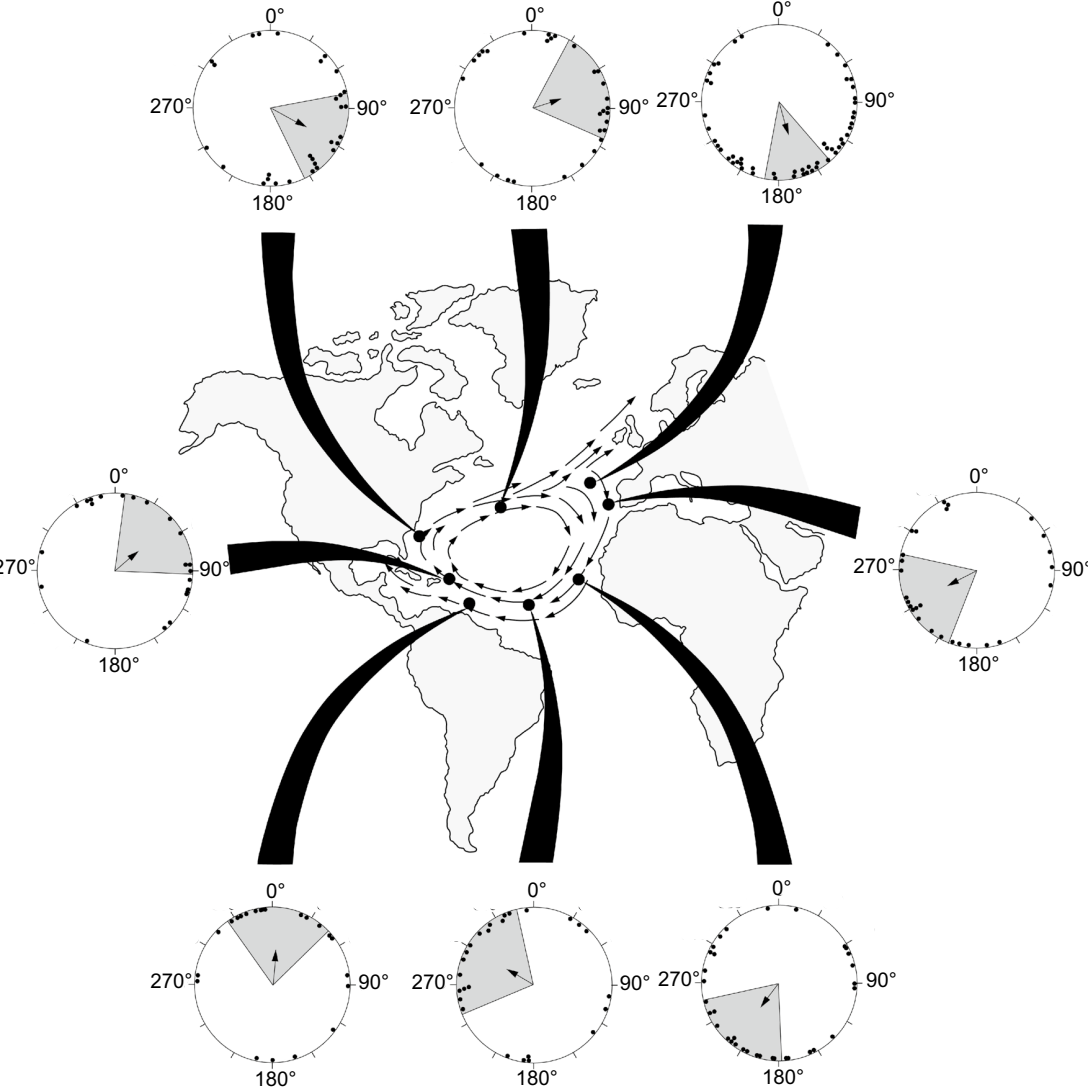
Orientation of hatchling loggerhead turtles in magnetic fields characteristic of widely separated locations along their migratory route in the North Atlantic Subtropical Gyre. The fields used in experiments replicated ones that exist at the locations on the map marked by black dots. Generalized main currents of the gyre are represented on the map by arrows. In the orientation diagrams, each dot represents the mean angle of a single hatchling. The arrow in the center of each circle indicates the mean angle of the group. The shaded sector represents the 95% confidence interval for the mean angle. Each group of turtles was significantly oriented at *p* < 0.05 or better. In each case, the direction of orientation was suitable for helping turtles remain in the gyre and advance along the migratory route. For details about the responses and why each is likely to be adaptive, see Lohmann et al. (2001, 2012), Fuxjager et al. (2011), Putman et al. (2011, 2015). Figure modified from Lohmann et al. (2012)

Subsequent experiments revealed that considerable information is encoded in the magnetic map of young loggerhead turtles (Lohmann et al. 2012). Turtles responded with oriented swimming to fields that exist in eight different locations along the migratory pathway, with the direction of orientation elicited by each field suitable for helping turtles remain in the gyre and advance along the migratory route (Fig. 2). Moreover, turtles can distinguish among locations that differ not only in the north–south (latitudinal) axis, but also in the east–west (longitudinal) axis. For example, when turtles were subjected to magnetic fields that exist at two different locations on opposite sides of the Atlantic Ocean—with the locations differing in longitude but not in latitude—the two fields elicited different responses (Putman et al. 2011). Hatchlings tested in the field from the eastern side of the Atlantic swam southwest, a direction consistent with the migratory pathway. By contrast, turtles tested in a field that exists near Puerto Rico, on the western side of the Atlantic, swam in a northeasterly direction likely to lead them into currents that facilitate rapid transport back to the US coast, where most Florida loggerheads spend their late juvenile years.

Prior to this study, a common view was that magnetic cues, which in most parts of the world vary more in a north–south direction than an east–west direction, are likely used by animals to determine latitude but not longitude (e.g., Mouritsen 2003; Alerstam 2006; Åkesson and Hedenström 2007; Gould 2008). The findings demonstrated, instead, that longitudinal information can be encoded into the magnetic map of an animal. At the same time, the results suggest that young sea turtles are indifferent to the human concepts of latitude and longitude and instead use magnetic signatures (i.e., pairings of inclination and intensity) to recognize important geographic areas and boundaries, where changes in swimming direction enhance survival (Lohmann et al. 2012).

#### Influence of ocean currents

The path of a young turtle is determined not only by the direction that it swims, but also by the direction that it is carried by ocean currents. In some oceanic regions, the velocity of currents greatly exceeds the swimming velocity of turtles (Revelles et al. 2007), raising the question of whether oriented swimming of hatchlings can have an impact on survival. Simulations with a high-resolution ocean circulation model provided evidence that small amounts of oriented swimming in response to regional magnetic fields—even as little as 1–3 h per day—greatly increase the likelihood of young Florida loggerhead turtles remaining safely within warm-water currents favorable for growth (Putman et al. 2012). These results are consistent with the interpretation that the magnetic map of young loggerheads plays a crucial role in helping turtles remain within the gyre system, advance along the migratory route, and avoid straying into dangerous areas.

Interestingly, fields from several locations along the migratory pathway have been found that do not elicit oriented swimming (Putman et al. 2015). Simulations using ocean circulation models suggest that, at these locations, drifting passively poses no danger to the turtles and, indeed, might sometimes promote retention in areas with abundant food (Putman et al. 2015). In light of this, it is perhaps not surprising that natural selection appears to have favored directional swimming only in response to magnetic fields that exist at locations where oriented movement promotes survival (Lohmann and Lohmann 1994; Merrill and Salmon 2011).

#### Magnetic maps and goal navigation in sea turtles

**Fig. 3:**
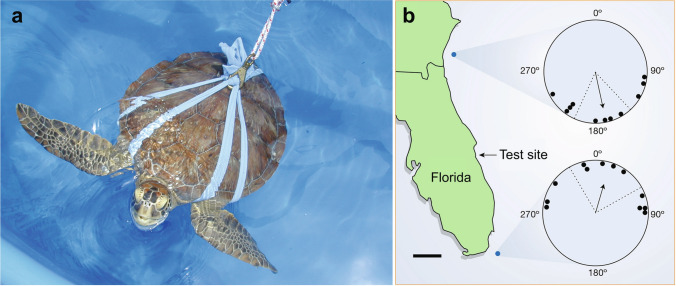
Evidence for a magnetic map in juvenile green turtles. **a** A juvenile green turtle swimming in a magnetic navigation experiment. Turtles were placed into soft cloth harnesses and tethered to an electronic tracking device that monitored their orientation as they swam in a water-filled arena surrounded by a magnetic coil system. **b** Juvenile turtles were captured in feeding grounds near the test site in Florida. Each turtle was exposed to a magnetic field that exists at one of two distant locations along the coastline (represented by the blue dots). Turtles exposed to the field from the northern site swam approximately southward, whereas those exposed to the field from the southern site swam approximately northward. In the orientation diagrams, each dot represents the mean angle of a single turtle. The arrow in the center of each circle represents the mean angle of the group. Dashed lines represent the 95% confidence interval for the mean angle. Map scale bar is 100 km. Figure modified from Lohmann et al. (2004)

In addition to the magnetic map inherited by young turtles, older sea turtles develop magnetic maps that can be used to facilitate navigation toward a particular location (Lohmann et al. 2004, 2007). After their initial long-distance migration through the open sea, juvenile sea turtles of several species take up residence in feeding grounds in coastal areas (Musick and Limpus 1997). Many turtles of this age show fidelity to specific foraging sites, returning to them after seasonal migrations and experimental displacements (Ireland 1980; Avens et al. 2003; Avens and Lohmann 2004). How turtles navigate to these foraging areas was studied using magnetic displacement of juvenile green turtles (*Chelonia mydas*) captured along the east coast of Florida. Turtles were placed individually into an orientation arena near the site of capture. Half were exposed to a magnetic field that exists at a location 337 km to the north; the other half were exposed to a field that exists at an equivalent distance to the south. Turtles subjected to the field from the northern location swam southward, whereas those subjected to the field from the southern location swam northward (Fig. 3). The turtles thus behaved as if they had been physically displaced to the two locations and were attempting to home from each site. The results demonstrate that, as they mature, sea turtles acquire a magnetic map that facilitates navigation toward specific goals. How turtles transition from the magnetic maps that guide hatchlings on their first migration to the magnetic maps used by older turtles to navigate to a goal is not known. One possibility is that the two systems are separate and independent, with hatchlings relying exclusively on responses they inherit and older turtles relying exclusively on information they have learned. Another possibility, however, is that the magnetic information inherited by hatchlings provides a critical foundation or framework that is filled in and perhaps expanded as turtles acquire a more extensive knowledge of magnetic topography through experience.

### Fish

Numerous fish follow lengthy migratory routes through the open sea and might, in principle, benefit from navigational mechanisms similar to those that exist in sea turtles. Although work on magnetic maps in fish began in earnest less than a decade ago, abundant evidence now exists that such maps are not only present among fishes, but widespread.

#### Inherited magnetic maps in fish

Many salmonid fish are anadromous. Adults spawn in streams and rivers; the offspring travel downstream to the river mouth, then migrate offshore to feeding grounds in the open sea. When the fish mature, they migrate back to their natal river system to spawn. Different species use different river systems as well as different oceanic areas as feeding grounds.

Several experiments have provided evidence that young salmon possess inherited magnetic maps. In an initial magnetic displacement experiment (Putman et al. 2014c), hatchery-reared Chinook salmon (*Oncorhynchus tshawytscha*) of the parr stage, which had never been in the ocean, responded to a magnetic field that exists close to the northern border of their range by orienting to the south-southwest. A second group exposed to a field from the southern border of the range oriented approximately north (Putman et al. 2014c). These results indicate that young salmon are able to respond to magnetic fields in the open sea before they ever enter the ocean. Had the fish actually been in the ocean, the responses presumably would have directed the salmon towards the center of their range and likely would have helped the fish remain in an oceanic area suitable for feeding.

**Fig. 4:**
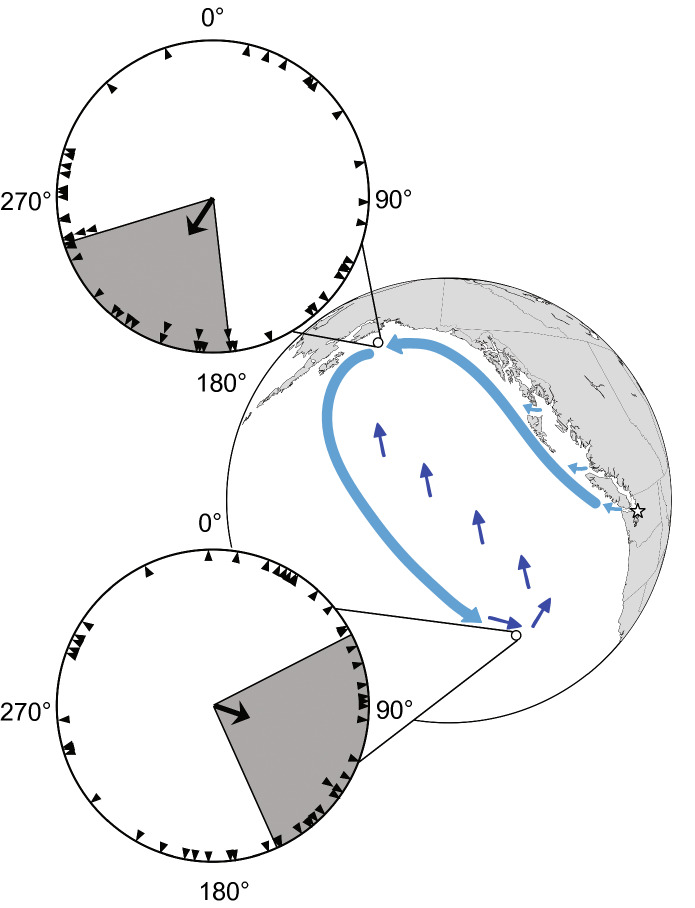
Orientation of juvenile pink salmon in response to magnetic fields that exist along the migratory route. The two long, curving, light-blue arrows through the Pacific indicate the migratory movements of pink salmon during their first year at sea. Short, dark-blue arrows in the open ocean show the presumed movements of fish during their second year; as adults, the fish migrate back to their coastal areas to spawn. Circular diagrams show the orientation of fish in response to the magnetic fields that exist at the two locations indicated on the map. Each triangle indicates the mean angle of a single fish. The arrow in the center of the circle indicates the mean angle of the group, with the shaded area representing the 95% confidence interval for the mean. Figure modified from Putman et al. (2020)

Another salmonid, the pink salmon (*Oncorhynchus gorbuscha*), likely uses magnetic positional cues to progress along its migratory path in a way that is reminiscent of sea turtle hatchlings (Putman et al. 2020). Juvenile pink salmon from the Pacific northwest follow an elliptical migratory route in which they move northward to Alaska, then southward through the open Pacific to areas near northern California, before eventually returning to their home region (Fig. 4). Hatchery-reared fish that had never been in the ocean, when exposed to a magnetic field that exists in Alaskan waters, oriented in a southwesterly direction consistent with the migratory route (Putman et al. 2020; Fig. 4). By contrast, fish exposed to a field from an offshore area near the southernmost part of their migration oriented east-southeast, again consistent with the migratory pathway (Fig. 4). A conceptually similar study with Atlantic salmon (*Salmo salar*) also yielded results suggesting that the fish use magnetic fields along their migratory route to guide their movements from the eastern U.S. to western Greenland (Minkoff et al. 2020). Interestingly, a landlocked population of fish descended from migratory Atlantic salmon also showed responses to regional magnetic fields in both the Atlantic and the Pacific, suggesting that responses to magnetic fields that might have been useful in an ancestral migration can persist for generations, even in fish populations that are prevented from migrating (Scanlan et al. 2018).

Magnetic displacement experiments have also revealed evidence for a magnetic map in other migratory fish like European eels (*Anguilla anguilla*). These fish reproduce in the Sargasso Sea, after which larval eels are transported by ocean currents associated with the Gulf Stream System to coastal and freshwater habitats from North Africa to Scandinavia (Schmidt 1923; Tesch et al. 1992). Fish at the glass eel stage, which had recently arrived in an estuary in the U.K., were exposed to magnetic fields replicating those that exist at several locations along or near their migratory route through the Atlantic (Naisbett-Jones et al. 2017). A magnetic field that exists near the Sargasso Sea breeding grounds elicited southwesterly orientation, while a field from the northwest Atlantic (off the northern U.S. east coast) elicited northeasterly orientation. Analyses with an ocean circulation model revealed that, at the two locations, swimming in the observed directions would be expected to increase the number of juvenile eels that enter the Gulf Stream System. Thus, the responses of eels likely facilitate transport by ocean currents, providing an energetically efficient route toward Europe. Magnetic fields from two locations closer to Europe failed to elicit oriented responses, but at these locations, currents appear likely to transport eels eastward toward European developmental habitats regardless of whether they swim. Thus, like sea turtle hatchlings (Putman et al. 2015), young eels apparently lack responses to magnetic fields that exist in places where passive drift poses no threat.

#### Magnetic maps and goal navigation in fish

Evidence consistent with a magnetic map used in goal navigation—one that might be either learned or inherited—has been reported in bonnethead sharks (*Sphyrna tiburo*) (Keller et al. 2021). Juvenile sharks were captured in the Gulf of Mexico near the Florida panhandle, where land prevents sharks from traveling north. Sharks exposed to a magnetic field that exists about 600 km south of their capture site oriented northward, but sharks exposed to a field that exists an equivalent distance to the north (and on land) oriented randomly. One possibility is that the sharks tested had learned how field parameters change to the south and could thus recognize a southern magnetic displacement, but lacking experience with northward travel, they could not recognize a simulated northern displacement. Alternatively, sharks from this population might have inherited a response to fields that lie to the south, but not to fields that exist on land where they never travel. Either way, the results provide evidence that bonnethead sharks have a magnetic map that facilitates return to a home area, while also highlighting the challenges of disentangling what information in such maps is inherited and what is learned.

### Birds

The autumn disappearance and sudden spring return of birds represents the first animal migration that Western science sought to investigate (Lohmann 2018). When research on the navigation of migratory birds began in earnest, rapid progress was made in identifying the various compasses that birds use (Wiltschko and Wiltschko 2003), but the positional information that birds exploit has remained more elusive. Nonetheless, recent experiments indicate that at least some birds have magnetic maps that help guide migratory movements.

#### Inherited magnetic maps in birds

Relatively few studies have investigated whether young birds migrating for the first time have inherited magnetic maps similar to those of turtles and fish, yet results suggest that such a map might exist in at least one population of the pied flycatcher, *Ficedula hypoleuca* (Beck and Wiltschko 1988). The central European population of pied flycatchers has a two-step migration that consists of first flying southwest to Iberia, then changing to a southeasterly course. This pattern of movement enables the birds to avoid major barriers by skirting the Alps, the Mediterranean Sea, and the central Sahara.

When captive flycatchers were exposed to a sequence of magnetic fields like one they encounter while migrating, they changed direction from southwest to southeast at the same time as birds normally do during the natural migration (Beck and Wiltschko 1988). Birds maintained in the local field at the migration start point did not change direction, nor did birds held in a field that exists near the migratory endpoint. Thus, the results suggest a complex interaction between magnetic parameters and an endogenous time program, in which the birds must experience fields that exist along the migratory route at appropriate times to orient correctly at each point in the migration (Beck and Wiltschko 1988). Interestingly, pied flycatchers from a different population in the eastern Baltic changed their orientation direction spontaneously when kept in the constant magnetic field of their home area (Kishkinev et al. 2006; Kishkinev and Chernetsov 2015), suggesting that differences may exist among populations.

Although not involving orientation responses, several additional studies have demonstrated that birds migrating for the first time extract positional information from Earth’s magnetic field. The thrush nightingale (*Luscinia luscinia)* migrates south across the Saharan desert, an immense region where food is scarce. Birds maintained in Sweden were exposed either to the local magnetic field or to a sequence of magnetic fields that exist along the migratory pathway towards the Sahara. Those animals that had experienced the simulated magnetic migration gained significantly more weight than control birds, suggesting that specific regional magnetic fields encountered during the migration trigger the accumulation of fuel needed to power the flight across the desert (Fransson et al. 2001; Kullberg et al. 2003).

In a subsequent study with wheatears (*Oenanthe oenanthe*), one group of juvenile birds was exposed to a magnetically simulated autumn migration from southern Sweden to West Africa, while another was exposed to fields simulating a parallel but unnatural flight out over the Atlantic (Boström et al. 2010). The second group increased fuel deposition relative to birds that experienced the simulated natural migration, consistent with the hypothesis that the birds used magnetic cues to assess their position and perceived the fields from the Atlantic as an indication of a longer-than-expected migration. The results reveal that birds, on their first migration, can use geomagnetic cues to compensate for a displacement outside their normal migratory route by adjusting fuel deposition. A different study with wheatears focused on migratory restlessness, the tendency of captive birds to show elevated levels of activity at times when they would normally be migrating. Restlessness increased strongly over the course of the migratory season when the birds were maintained in the magnetic field of northern Germany, but decreased when the birds were subjected to magnetic field changes that exist along the birds’ natural flyway (Bulte et al. 2017). All these findings are consistent with the interpretation that birds derive ‘map’ information from the magnetic field and use it to optimize their migrations (Heyers et al. 2017). The ability of first-time migrants to compensate for natural or experimental displacements under some conditions (e.g., Åkesson et al. 2005; Thorup and Rabøl 2007; Thorup et al. 2011) is also consistent with the use of inherited magnetic map information, although alternative explanations might also exist.

#### Magnetic maps and goal navigation in birds

**Fig. 5:**
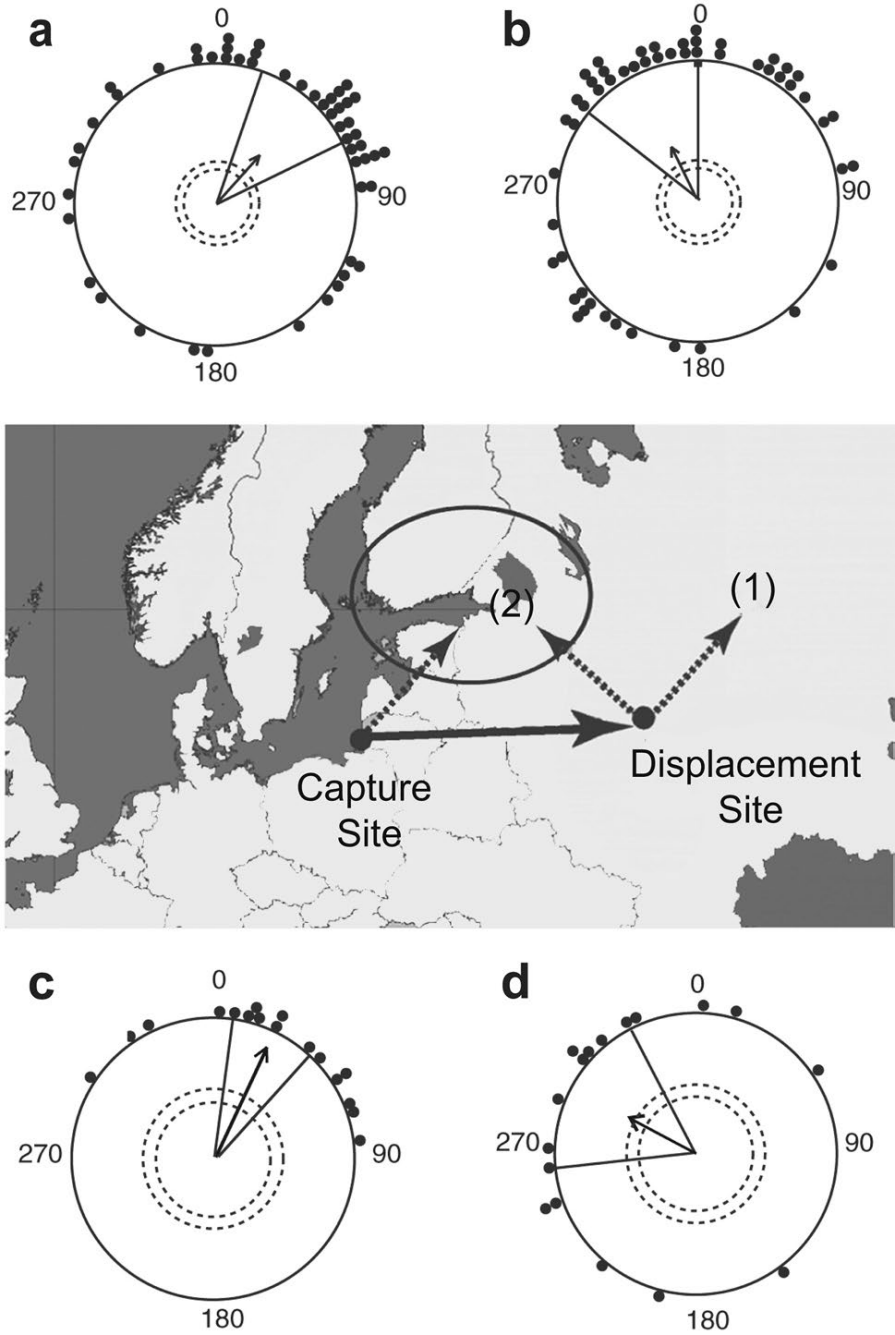
Evidence for a magnetic map in the Eurasian reed warbler. Map depicts location of the capture site and the site to which birds were displaced (physically in one experiment and by magnetic displacement in another). The capture and displacement sites were approximately 1000 km apart. The breeding destination of the birds is circled in the center. The broken arrow at the capture site indicates mean migratory direction of Eurasian reed warblers passing through the location and the broken arrows at the displacement site represent two possible outcomes following displacement: (1) no compensation for displacement or (2) compensation for displacement, shown by re-orientation towards the breeding destination. Circular diagrams (top) show the orientation of birds during spring migration in 2004–2007 (Chernetsov et al. 2008). **a** Orientation at the capture site. **b** Orientation at the displacement site after birds were moved there physically. Circular diagrams (bottom) show the orientation of birds during spring migration in 2012–2013 (Kishkinev et al. 2015). **c** Orientation at the capture site. **d** Orientation in response to the magnetic field that exists at the displacement site. Both the physical displacement and the magnetic virtual displacement resulted in similar compensatory orientation towards the breeding destination. Each dot indicates the mean orientation of an individual bird. Arrows indicate mean angles for each group, with circles in the center of each circular diagram indicating the magnitude of the group mean vector required for 5% and 1% significance levels using a Rayleigh test for uniformity. Lines on either side of the arrows represent 95% confidence intervals for group mean directions. Figure modified from Kishkinev et al. (2015)

Evidence for a magnetic map used in goal navigation has been acquired in the Eurasian reed warbler (*Acrocephalus scirpaceus*). In initial experiments, birds were captured during spring migration at a location along the southeastern Baltic coast where they normally migrate northeast to reach their breeding sites (Fig. 5). Control birds tested at the capture site oriented in a northeasterly direction as expected. In contrast, birds physically displaced 1000 km eastward oriented in a northwesterly direction, implying that the birds detected the displacement and compensated for it (Chernetsov et al. 2008; Kishkinev et al. 2013). In a subsequent magnetic displacement experiment (Kishkinev et al. 2015), birds were not physically moved from their capture area, but instead were exposed to the magnetic field of the location 1000 km to the east. The birds showed re-orientation indistinguishable from that elicited by physical displacements in the earlier experiments, confirming that reed warblers have a magnetic map that facilitates navigation toward their breeding area (Fig. 5).

Findings suggest that the magnetic map of at least some birds relies partly on magnetic declination—the difference between true geographic north and magnetic north. Detection of true north requires monitoring the movement of stars during the night, which in turn allows identification of the geographic pole; stars above the poles remain stationary while the rest of the night sky appears to rotate. Given that many birds have a ‘star compass’ based on this principle, the idea that birds detect declination is plausible (Wiltschko and Wiltschko 2003; Åkesson et al. 2005). Declination is a less likely cue for aquatic animals such as sea turtles and fish, which migrate below water under conditions that usually preclude viewing the night sky.

Evidence consistent with the hypothesis that birds detect declination was reported in experiments with migratory white-crowned sparrows (*Zonotrichia leucophrys gambelii*) in the high Arctic of North America (Åkesson et al. 2005). Adult and juvenile birds were physically displaced eastward aboard a ship and their orientation was monitored at various locations 266–2862 km from their capture site. After displacement across the 0° declination line, both adults and juveniles abruptly shifted their orientation from the migratory direction to a direction leading back toward the breeding area and normal migratory route. One possible interpretation is that the birds used declination, perhaps in combination with celestial cues, to correct for longitudinal displacements.

Additional evidence that birds sense declination was reported in a study with reed warblers (Chernetsov et al. 2017). Adult birds exposed to a declination existing at a location west of their capture site oriented eastward, consistent with the hypothesis that the birds use declination to assess position. In similar studies with two other species of migratory bird, however, the same changes in declination failed to elicit a change in orientation, suggesting either that not all birds detect declination, or that different species use it (or weight it) in different ways (Chernetsov et al. 2020). To complicate matters, a subsequent study with reed warblers reported that birds did not respond to a change in declination alone, but did respond to a magnetic displacement in which all parameters of the field (inclination, intensity, and declination) were changed together (Kishkinev et al. 2021). At first glance, these results appear to contradict earlier ones (Chernetsov et al. 2017), but differences in the two experiments may explain the disparate outcomes. In the first study, the declination-only change may have simulated displacement to a real location, whereas in the second, the combination of magnetic parameters used in the declination-only change does not exist in nature, perhaps causing the birds to preferentially weight inclination and intensity in this situation (Kishkinev et al. 2021). Of course, another possibility is that birds usually rely on inclination and intensity in much the same way that turtles and fish do, and that declination is unimportant in most settings. An answer awaits future experiments.

These considerations notwithstanding, the overall results provide evidence for an avian magnetic map that facilitates navigation toward a breeding area. Similar maps appear likely to exist in numerous bird species; indeed, evidence to this effect has begun to accrue (Fischer et al. 2003; Henshaw et al. 2010; Deutschlander et al. 2012; Holland and Helm 2013). The possibility that homing pigeons navigate using magnetic maps has also long been debated, but no consensus has yet emerged among those who study them (Gould 1982; Wiltschko and Wiltschko 1995; Wallraff 1999, 2005; Papi 2001; Dennis et al. 2007; Gagliardo et al. 2009; Wiltschko et al. 2010; Gould and Gould 2012). A possible factor influencing variation in results with homing pigeons is that these birds have been selectively bred to be extraordinary navigators; thus, they detect numerous cues and flexibly switch among them (Walcott 1996). In light of this, pigeons might plausibly exploit magnetic cues as part of a map in some geographic areas and settings, but not in others (Beason and Wiltschko 2015; Walcott et al. 2018).

### Lobsters

Although most research on magnetic maps has focused on long-distance migrants such as sea turtles, salmonids, and birds, such maps also exist in animals that move over considerably shorter distances. The Caribbean spiny lobster, *Panulirus argus*, is a migratory crustacean indigenous to the Caribbean and the southeastern U.S.A. During the summer lobsters hide in crevices and holes during daylight hours, but at night they emerge to forage over a considerable area before returning in darkness to the same den or another one nearby (Herrnkind and McLean 1971; Herrnkind 1980). The lobsters have a remarkable homing ability and can orient toward capture areas even when displaced to unfamiliar sites and deprived of all known orientation cues *en route* (Creaser and Travis 1950; Boles and Lohmann 2003). Indeed, the spiny lobster is the only invertebrate presently known to fulfill the criteria of true navigation, defined as the ability to determine position relative to a goal in an unfamiliar area, without using cues associated with the destination or information obtained during the outward journey (Boles and Lohmann 2003).

**Fig. 6:**
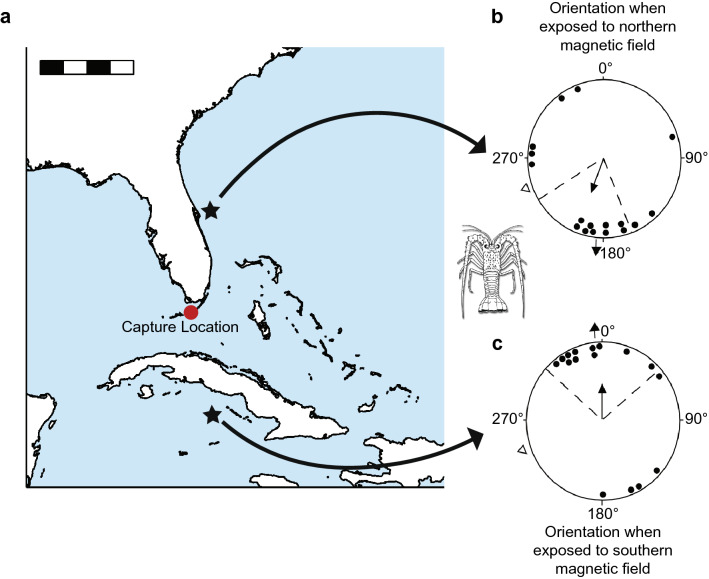
Evidence for a magnetic map in spiny lobsters. The diagram shows orientation of lobsters captured in the Florida Keys and tested in magnetic fields replicating those that exist at two different geographic locations (marked by stars on the map). Lobsters tested in a field replicating one that exists north of the test site walked southward, whereas those tested in a field like one that exists south of the test site walked northward. The arrow outside of each diagram indicates the direction in which lobsters would be expected to orient if they were homing from the locations indicated by the stars. The open triangle outside each orientation diagram indicates the actual direction to the capture site from the test site. In each case, lobsters responded as if they had been displaced to the locations marked by the stars rather than by orienting in the direction that was actually towards the capture site. Scale bar = 400 km. Figure modified from Boles and Lohmann, 2003

In magnetic displacement experiments, spiny lobsters exposed to a field that exists north of the capture site oriented southward, whereas those tested in a field replicating one that exists an equivalent distance to the south oriented northward (Fig. 6) (Boles and Lohmann 2003). These results demonstrate that spiny lobsters possess a magnetic map that facilitates navigation toward specific areas where they can find shelter.

### Newts

Although spiny lobsters sometimes travel distances of up to 200 km (Herrnkind 1980), perhaps the most surprising finding about magnetic maps is that one may exist in salamanders that never travel farther than a few kilometers. Red-spotted newts (*Notophthalmus viridescens*) displaced 23–42 km from a home pond, under conditions that presumably made following the outward journey impossible, were nevertheless able to orient back toward the capture site (Phillips et al. 1995). As described previously, newts tested in a magnetic field with an inclination angle found to the north oriented southward, while newts exposed to an inclination angle found to the south oriented northward (Fischer et al. 2001; Phillips et al. 2002).

One caveat is that the newt studies paired an intensity found in a home area with an inclination that exists to the north or south, so that the magnetic fields used do not precisely match any that exist in the natural habitat. Experiments with birds (e.g., Kishkinev et al. 2021), as well as studies with turtles (Lohmann and Lohmann 1994; Lohmann et al. 2012), suggest that changing a single magnetic parameter alone does not always elicit the same response as a magnetic field fully replicating one that exists at an actual location. Thus, a useful future step will be to determine how newts respond to fields that exist in their environment. The small distances that newts move, the extremely small field changes that they presumably experience, and the presence of small daily fluctuations in the earth’s field—referred to as solar quiet day variation or *S*_q_ (Skiles 1985)—have all been raised as potential obstacles to using a magnetic map over short distances (Kishkinev and Chernetsov 2015; Komolkin et al. 2017; Mouritsen 2018). Nevertheless, the fact remains that several species of newt home after short-distance displacements (Phillips et al. 1995; Sinsch 2006). Thus, one way or another, these animals seem to assess where they are relative to a nearby goal.

Recent experiments with the Alpine newt (*Ichthyosaura alpestris*) have suggested a possible strategy that newts and other animals might use to more accurately sense slight differences in magnetic fields that exist between locations separated by only a few kilometers (Diego-Rasilla and Phillips 2021). Newts displaced from breeding ponds to new locations 4–9 km away oriented toward the capture site if they were held at the new site overnight before testing, but not if they were transported to the testing site on the day of testing. Moreover, newts held overnight at a ‘false testing site’, but then tested at a different location, oriented in the direction that led home from the holding site, but not from the site where they were actually tested. Additional results suggested that the critical determination of position was made around sunset. Although the experiments did not directly demonstrate that newts used magnetic cues to assess their position, the findings raise the intriguing possibility that newts measured the ambient magnetic field during evening twilight, a time when the temporal variation in Earth’s magnetic field is usually minimal (Diego-Rasilla and Phillips, 2021).

## Magnetic maps, natal homing, and geomagnetic imprinting

Many of the most extraordinary feats of animal navigation involve animals migrating long distances to return to an area of origin to reproduce. For example, some sea turtles, fishes, birds, and marine mammals travel immense distances before eventually navigating back to the approximate area—or sometimes even the precise location—where they began life (Meylan et al. 1990; Lohmann et al. 2008c; Rooker et al. 2008; Welch et al. 2012; Baker et al. 2013; Feldheim et al. 2014). Although this pattern of behavior exists in diverse animals, the terminology used to describe it varies among biologists studying different animal groups. For sea turtles, the term *natal homing* has long been used; for salmon and other migratory fish, the simpler term *homing* is more common, and for birds, the terms *philopatry* or *natal philopatry* are sometimes preferred. Of the various terms, philopatry is perhaps the least precise, inasmuch as the term can be used to refer to animals that either remain in an area or return after a migration (Hendry et al. 2004). When used in the context of migratory animals, however, the terms homing, natal homing, and philopatry are functionally equivalent, in that all denote return to an area of origin after first migrating a long distance away.

The concept of long-distance natal homing based on a magnetic map sense and imprinting was initially developed in the context of sea turtles and salmon (Lohmann and Lohmann 1994; Lohmann et al. 1999, 2008c). In its simplest form, the hypothesis proposes that young animals imprint on the magnetic field of their natal area (Box 3), then use this information to navigate back to the region using a magnetic map as adults (Lohmann et al. 2008c, 2013). In the past decade, strong evidence consistent with this idea has rapidly accumulated in several animals including salmon (Putman et al. 2013, 2014a), sea turtles (Brothers and Lohmann 2015, 2018), seabirds (Wynn et al. 2020), sharks (Keller et al. 2021), and insects (Oh et al. 2020). Indeed, the emerging picture suggests that a magnetic map sense, combined with imprinting-like learning of the magnetic field of a home region, represents a widespread solution for long-distance natal homing among migratory animals (Lohmann and Lohmann 2019). Next, we summarize recent developments in this area.

Box 3: Imprinting Imprinting is a special form of learning. Although precise definitions of imprinting vary (e.g., Hasler and Scholz 1983; Goodenough et al. 2010; Zupanc 2010; Alcock 2013), the hallmarks of imprinting are that the learning occurs during a specific, critical period (typically early in the life of an animal), the effects are long lasting, and the learning is not easily modified. The geomagnetic imprinting hypothesis of natal homing (Lohmann et al. 2008c) proposes that animals learn the magnetic field of their area of origin when young, then use this information in combination with a magnetic map sense to return as adults. The learning process might or might not meet the strict ethological definition of imprinting, depending on the animal. For example, long-lived animals such as sea turtles and birds, which reproduce in multiple years, might update their knowledge of the magnetic field of a nesting area each time they visit (Lohmann et al. 1999, 2008c, 2013; Gould, 2015), rather than learning the field only on a single occasion when they are young.

**Natal homing in sea turtles**

**Fig. 7:**
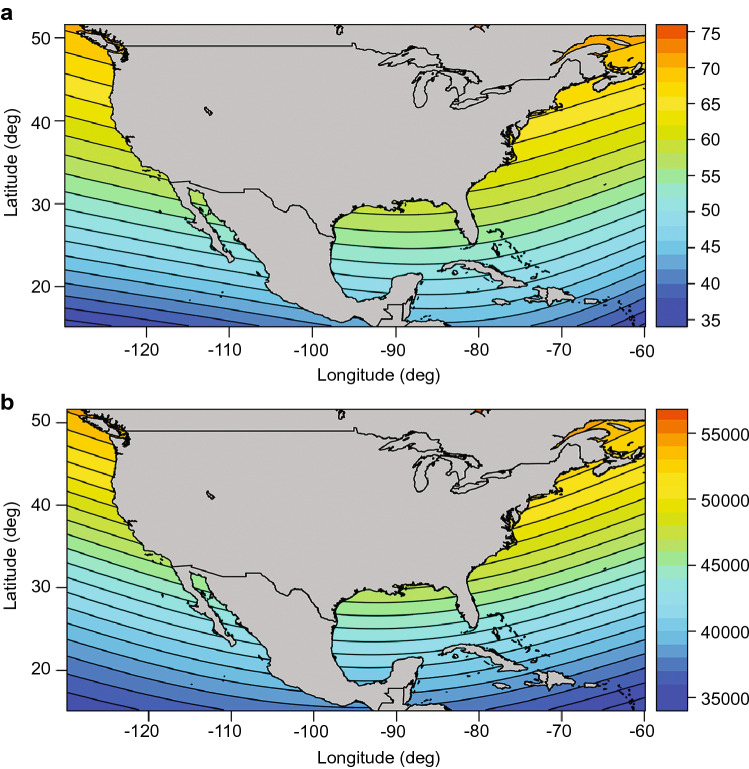
Magnetic isolines along the coasts of North America. **a** Isolines of magnetic field inclination. Black isolines bordering each color on the map indicate increments of 2°. Color scale to the right of the map indicates inclination angle in degrees. **b** Isolines of total field intensity. Black isolines bordering each color on the map indicate increments of 1,000 nT. Color scale to the right of the map indicates total field intensity in nT. Note that each region of the east and west coast is marked by a different inclination angle and intensity. Isolines were derived from the International Geomagnetic Reference Field (IGRF) model 12 for 2018 (Thébault et al. 2015)

Most major sea turtle rookeries are located along continental coastlines that trend north–south; isolines of magnetic intensity and inclination in these areas, however, trend east–west (Lohmann et al. 1999, 2008c). Thus, each coastal area is marked by a unique magnetic signature (Fig. 7). A simple navigational strategy for returning to a natal site might consist of a turtle swimming along the coastline until it encounters magnetic parameters remembered from its area of origin (Lohmann et al. 2008c; Lohmann and Lohmann 2019). In principle, a turtle might seek out a particular intensity, inclination, or combination of both.

**Fig. 8:**
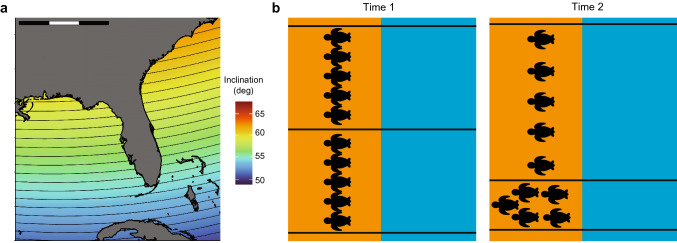
Predicted changes in nesting density in response to movement of magnetic isolines if turtles identify natal sites using magnetic signatures. **a** Map of Florida showing isolines intersecting both the Atlantic and Gulf coasts of the peninsula. Black isolines indicate increments of 0.5°. Isolines were generated for 2015 using IGRF model 13; isolines of intensity also intersect both coasts but are not shown. Scale bar = 600 km. **b** Effect of isoline movement on the return of turtles to the natal beach. Blue indicates ocean and tan indicates beach. Horizontal lines represent three magnetic isolines. Each turtle represents a nesting female that has imprinted as a hatchling on the magnetic signature that marked its natal site. At Time 1, turtles nest with equal density in two areas of the beach. When the turtles return to nest again several years later at Time 2, the isolines have moved due to secular variation (see Box 2). The top two isolines have diverged while the bottom two isolines have converged. At times and places where isolines diverge, the geomagnetic imprinting hypothesis predicts a decrease in nesting density, because turtles that imprinted on the fields between the isolines should return to nest over a larger area. In places where isolines converge, the hypothesis predicts that nesting density should increase, because turtles will return to nest over a shorter span of coastline. Figure modified from Brothers and Lohmann (2015)

If turtles identify their natal beaches in this way, then subtle changes in Earth’s magnetic field might affect the distribution of turtle nests (Brothers and Lohmann 2015). Due to secular variation, magnetic isolines gradually shift position, but the direction and distance that an isoline moves along a coast varies among locations and years. At some times and in some locations, isolines intersecting the coastline edge closer together so that the distance between them becomes smaller (Fig. 8). Under these conditions, if female turtles returning to nest seek out the magnetic signatures that mark their natal beaches, then they should nest along a shorter length of coastline, and the density of nests (number per unit distance) should, therefore, increase (Fig. 8b). By contrast, isolines along the coast sometimes move apart so that the distance between them becomes larger. When this happens, nesting density would be expected to decrease, because turtles returning to nest would be expected to select sites distributed over a greater length of coastline (Fig. 8b). An analysis of a 19-year database of sea turtle nesting along the Florida east coast confirmed both predictions (Brothers and Lohmann 2015), providing evidence that turtles do indeed locate their natal beaches by seeking out specific magnetic signatures.

**Fig. 9:**
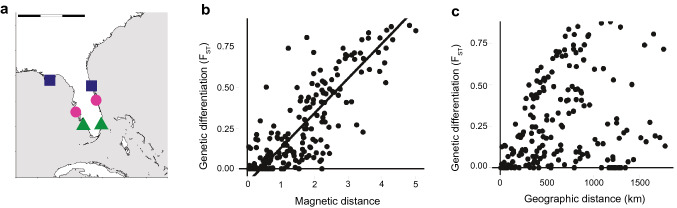
Analyses of population structure of loggerhead turtles in the southeastern U.S. in the context of magnetic parameters. **a** Genetic analysis of nesting turtles along the east and west coasts of Florida (Shamblin et al. 2011) suggested an unusual pattern in which populations at similar latitudes but on opposite coasts had similar haplotype frequencies; for example, the east coast population marked by the dark-blue square is more similar genetically to the west coast population marked by a dark-blue square than it is to the closely adjacent east coast population marked by the pink circle. This is potentially significant because beaches at similar latitudes on opposite sides of the Florida peninsula have similar magnetic signatures (see Fig. 8a). **b** Relationship between F_ST_ and magnetic distance. Each data point results from a pairwise comparison between nesting beaches in the southeastern U.S. where nesting turtles were sampled. F_ST_ represents pairwise comparisons of genetic differentiation; a low F_ST_ indicates high genetic similarity. Magnetic distance is a metric reflecting the difference in magnetic fields between two nesting locations (Brothers and Lohmann 2018); low magnetic distance indicates that the magnetic fields at two beaches are very similar, while a high magnetic distance implies a large difference in magnetic fields. A strong positive relationship exists between magnetic distance and genetic differentiation (*p* = 0.001). Turtles nesting on beaches with similar magnetic fields tend to be genetically similar, whereas turtles that nest on beaches with different magnetic fields tend to be genetically dissimilar. **c** Relationship between F_ST_ and geographic distance. No significant relationship was found between these two parameters. Data are from Brothers and Lohmann (2018)

Studies of population genetics have provided additional evidence consistent with the hypothesis that geomagnetic imprinting and magnetic maps underlie natal homing (Shamblin et al. 2011; Lohmann et al. 2013; Brothers and Lohmann 2018). Analyses revealed that populations of loggerhead turtles that nest on opposite sides of the Florida peninsula, but at similar latitudes, have similar haplotype frequencies (Fig. 9a) (Shamblin et al. 2011). This finding is of interest because the magnetic fields at latitudinally similar locations on opposite sides of Florida are similar (Fig. 8a), despite the long geographic distance between them. Thus, an interesting possibility is that population structure of sea turtles in the southeastern U.S.A. has arisen partly because of errors in magnetic navigation during natal homing. For example, turtles seeking out the magnetic signature of their natal beach along the Florida east coast might occasionally stray into the Gulf of Mexico and mistakenly nest on a different beach with a similar magnetic signature (Shamblin et al. 2011; Lohmann et al. 2013).

The population structure of loggerhead turtles in the southeastern U.S.A. was analyzed in the context of magnetic fields that exist at different nesting beaches (Brothers and Lohmann 2018). The analysis used values of *F*_*ST*_, a metric ranging from zero to one in which low values indicate genetic similarity between two populations and high values indicate genetic differentiation. *F*_*ST*_ values were obtained from pairwise comparisons between all possible combinations of nesting beaches where turtles had previously been sampled. For each pair of beaches, the difference between the magnetic fields at the two locations was also calculated, as were metrics of geographic distance and environmental similarity.

A striking relationship emerged between spatial variation in Earth’s magnetic field and genetic differentiation (Fig. 9b, c). Turtles nesting at beaches with similar magnetic fields tended to be genetically similar, whereas turtles nesting at beaches with greater differences in magnetic fields had larger genetic differences. This relationship held even when geographic distance and environmental similarities were considered. These results mirror what would be expected if turtles imprint on the magnetic field of their natal areas and seek out the same magnetic signature along the coastline when they return to nest as adults, but occasionally mistake other beaches with similar magnetic fields for the intended destination.

Additional evidence consistent with turtles using magnetic cues to navigate to nesting areas was obtained in a study in which adult turtles were captured on an island and physically displaced to locations 100–120 km away, after which they were tracked with satellite telemetry (Luschi et al. 2007). Turtles with strong magnets attached to their heads returned to the island by more indirect routes, as well as more slowly, than control turtles. These results indicated that disrupting the magnetic field around turtles impaired their navigation, but whether the effect was on a magnetic map, a magnetic compass, or both could not be determined (Luschi et al. 2007; Lohmann et al. 2008b).

**Natal homing in salmon**

Salmon are known to imprint on olfactory cues of their home rivers and use this information to help them return to specific spawning areas (Hasler and Scholz 1983; Dittman et al. 1996). During their reproductive migration, however, salmon of many populations must first navigate from the open sea to the proximity of their specific river, a process that is unlikely to be mediated by olfactory cues given the distances involved (Quinn 2018). In principle, salmon might imprint on the magnetic field of the area where they first enter the sea, and thus use magnetic information to arrive in the vicinity of the target river—close enough that they can use chemical cues to find the river itself (Lohmann et al. 2008a, 2008c).

**Fig. 10:**
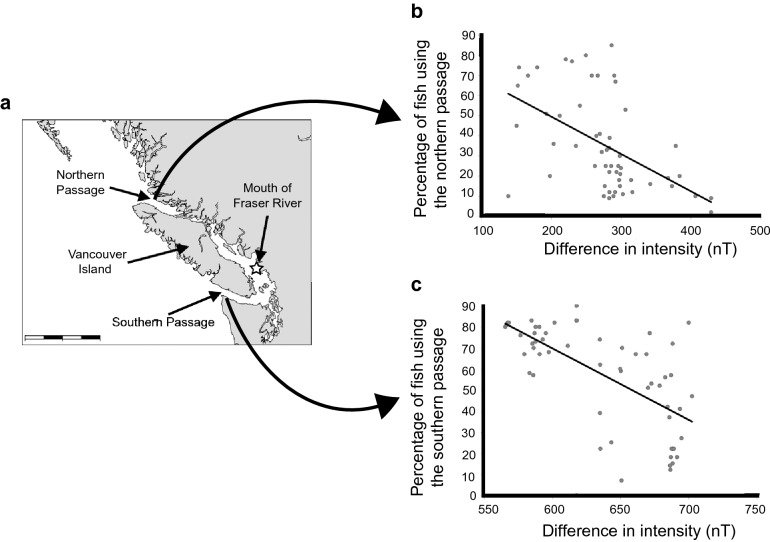
Evidence that salmon navigate into the proximity of their natal rivers using magnetic cues. **a** Map of Vancouver Island showing the northern and southern routes that Pacific salmon can follow to reach the Fraser River during spawning migrations. Scale bar = 225 km. **b** Percentage of fish using the northern route in relation to the difference between the magnetic intensity of the Fraser River mouth when the fish departed the river and the magnetic intensity of the northern passage when they returned. Each data point represents one year. The percentage using the northern route declined as the difference in magnetic intensity increased. **c** Percentage of fish using the southern route in relation to the difference between the magnetic intensity of the river mouth when the fish departed and the magnetic intensity of the southern passage when the fish returned. The percentage using the southern route declined as the difference in magnetic intensity increased. Data were derived from Putman et al. (2013)

Indirect yet strong evidence for geomagnetic imprinting in salmon came from two studies involving large, multi-year datasets of the migratory routes followed by sockeye (*Oncorhynchus nerka*) and pink salmon returning from the Pacific to spawning sites in the Fraser River. Fish cannot swim directly to the river mouth because Vancouver Island blocks the way; thus, they must detour around the island through one of two passageways, one northern and one southern (Fig. 10) (Putman et al. 2013). If salmon imprint on the magnetic field when leaving the river mouth, then on their return migration, fish would be expected to choose the passageway with the magnetic field that most closely resembles the field on which they imprinted. Indeed, the more the magnetic field at a passageway drifted from the magnetic field that existed at the river mouth when the fish departed, the fewer fish used that passageway upon return (Fig. 10). When information on magnetic field changes was incorporated into models designed to explain variation in the migratory routes taken by sockeye and pink salmon, performance of the models improved significantly (Putman et al. 2014a). Taken together, these studies provide strong circumstantial evidence that salmon imprint on the magnetic field of their home area and use this information to navigate back to the vicinity of the river mouth (Putman 2018; Lohmann and Lohmann 2019).

**Natal homing in seabirds**

**Fig. 11:**
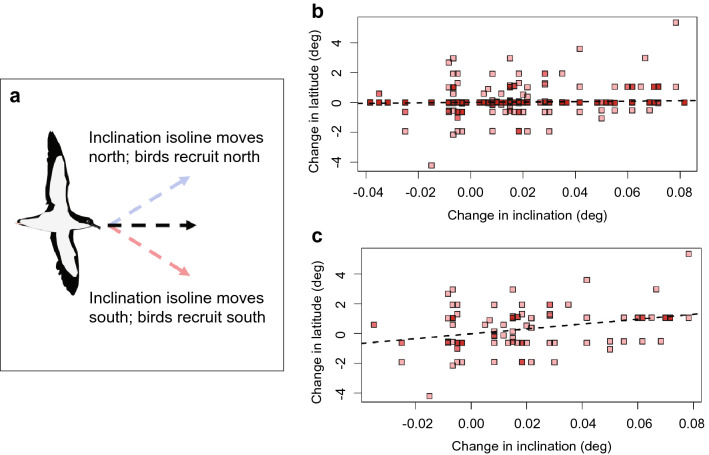
Recruitment latitude for fledgling shearwaters that returned to a non-natal colony corresponds with changes in magnetic inclination during absence. **a** Predicted change in recruitment latitude if birds imprint on the magnetic inclination of their nesting site and use this information to return to a coastal location. If inclination decreases in the area, then birds should recruit north of their natal colony (indicated by blue arrow). If inclination increases, birds should recruit south of their natal colony (indicated by red arrow). Black arrow represents accurate recruitment to natal location. **b** Change in inclination plotted against change in recruitment latitude for all fledgling birds. When all birds (*n* = 2996) were considered together, no significant relationship existed between the two parameters. **c** Change in inclination plotted against change in recruitment latitude for fledgling birds that changed location. Most birds recruited to natal locations, but for those that did not (*n* = 109), a relationship existed between the change in inclination angle and the direction/distance that they moved. Figure modified from Wynn et al. (2020)

A recent study with Manx shearwaters (*Puffinus puffinus*) provides evidence that the principles of geomagnetic imprinting and magnetic navigation uncovered in sea turtles and salmon are also relevant to sea birds. Wynn et al. (2020) used 80 years of bird ringing data to investigate whether shearwaters use magnetic cues to relocate their coastal breeding grounds. Manx shearwaters nest on northern European islands, but travel thousands of kilometers to the coast of Argentina to forage. Birds were ringed before their first migration, so that the site of the nest was known and birds could be identified individually upon return. Although most birds returned to the immediate vicinity of the site where they were raised, a subset of birds changed location. The observed changes in location were strongly correlated with changes in inclination angle that occurred between the time when the bird fledged and when it returned three years later to breed for the first time (Fig. 11) (Wynn et al. 2020). Interestingly, younger birds were more likely to shift breeding colonies than older birds. This might indicate that, as birds’ age, they gain experience and become more adept at using other navigational cues to relocate their breeding site. The results are consistent with the hypothesis that shearwaters imprint on the magnetic field of their natal area and use this information to return, a finding that may be relevant to numerous other bird species. Overall, the emerging evidence that sea birds, turtles, and fishes all use similar principles to return to their natal areas suggests that navigation with a magnetic map sense underlies natal homing in numerous animals.

**Geomagnetic imprinting in insects**

Surprisingly, evidence consistent with magnetic imprinting has also been acquired in the fruit fly *Drosophila* (Oh et al. 2020). Fruit flies are not known to migrate long distances, but flies do sometimes travel at least 10–15 km (Jones et al. 1981; Coyne et al. 1982). Early in development, flies were exposed to one of three magnetic fields matching those that exist in different, widely separated geographic areas. Later, as adults, hungry flies responded to the field that they had experienced previously, but not to other fields, by moving downward, a geotactic behavior associated with foraging (Oh et al. 2020). These results provide experimental evidence that organisms can learn and remember a magnetic field to which they were exposed during a critical period of development. Although the function of this behavior in fruit flies is not known, one possibility is that imprinting on the magnetic field of a natal area assists flies and their offspring in recognizing locations likely to be favorable for foraging and reproduction. Whether flies can use the imprinted information to navigate toward specific locations has not yet been investigated.

## Organization and structure of magnetic maps

Despite the immense progress of the past 2 decades, little is known about exactly what information a magnetic map sense provides and how magnetic maps are structured. Indeed, the term ‘map’ itself, although ingrained in the animal navigation literature, is perhaps unfortunate, inasmuch as it brings to mind specific spatial representations used by humans (Walcott 1996). In reality, little is known about whether internal spatial representations exist in migratory animals and, if they do, how closely they resemble human conceptions of maps (Bennett 1996). Although it is conceivable that some animals form a mental image of their environment and associated magnetic topography and can place themselves on a cognitive representation of this magnetic landscape, there is no evidence yet that any animal does this. Instead, the information that a map sense provides might be limited to a direction that must be traveled. Even in the case of goal navigation, an animal might ‘know’ only that it is north of its destination, but not by how far.

A reasonable starting point is to try to identify magnetic parameters that are used in magnetic maps, but even here, our knowledge is limited and incomplete. In turtles and fish, the magnetic map sense appears likely to be based on inclination and intensity (Lohmann and Lohmann 1994, 1996a; Lohmann et al. 2012; Putman et al. 2014c). Newts are known to detect inclination (Fischer et al. 2001; Phillips et al. 2002), but whether they detect other magnetic parameters is unknown. In birds, declination might play a role in some species, although apparently not in all (Åkesson et al. 2005; Chernetsov et al. 2017, 2020; Kishkinev et al. 2021).

The emerging picture is that there is unlikely to be a universal magnetic map used by all animals, either in terms of magnetic parameters used or in the way that the map is structured. This is perhaps not surprising, inasmuch as evolution is opportunistic in shaping navigational strategies, with natural selection favoring whatever enables an animal to complete a given navigational task within a particular geographic area. Indeed, the pattern of variation in magnetic field elements differs greatly among different parts of the world. In some geographic regions, for example, isolines of inclination and intensity are aligned almost perpendicularly, so that each location is marked by a unique magnetic signature consisting of a specific combination of inclination and intensity; in others, the isolines of these two parameters are nearly parallel (Lohmann et al. 1999, 2007; Boström et al. 2012). Similarly, the potential utility of declination as a navigational cue varies widely with global location. These considerations suggest that different parameters may be useful in different geographic areas and that a magnetic map might provide varying levels of information in different places. In addition, some but not all navigational tasks can be accomplished using a single magnetic parameter such as inclination or intensity. Thus, the amount of positional information that can be extracted from the geomagnetic field varies with geographic region, and the optimal strategy for navigation probably varies with the task.

How then should we envision magnetic maps? How are they structured, what information is encoded, and what magnetic navigational strategies do animals use as they guide themselves through their environment? Here, we outline just a few of many possible ways that animals might use a magnetic map sense in navigation.

### Possible structure of magnetic maps

#### Bicoordinate magnetic maps

Discussions of magnetic maps have often focused on the possibility that animals incorporate two different magnetic field elements into a kind of all-purpose, bicoordinate map that can simultaneously guide migrations and enable animals to determine location relative to a goal (e.g., Gould 1985; Lohmann and Lohmann 1996a; Phillips 1996; Åkesson and Alerstam 1998; Freake et al. 2006). The idea of bicoordinate maps presumably resonates strongly with researchers in part because the concept so closely parallels our own spatial system of latitude and longitude, in which each point on the map is defined by unique combinations of the two coordinates.

Given that some animals appear to use combinations of inclination and intensity to identify particular locations (Lohmann et al. 2001, 2012; Putman et al. 2014c), it is conceivable that some animals navigate by continuously monitoring these two parameters, much as a human GPS can continuously monitor latitude and longitude and use these to compute a path to a goal. A simpler possibility, however, is that animals assess the pattern of variation, or gradient, of each parameter individually so that their bicoordinate map consists of two separate magnetic gradients (Lohmann et al. 2007). In principle, if an animal knows the magnetic inclination and intensity that exist at a goal, and if the isolines of the two parameters are not parallel in the geographic region, then the animal can potentially reach the destination by alternately using first one gradient and then the other. Although the path to the goal might be longer and less efficient than the most direct and linear route, such a strategy requires no cognitive representation of the environment, nor any special computational skills.

#### Gradient maps and true navigation

Consideration of bicoordinate maps often leads to discussion of the ‘gradient hypothesis’ (e.g., Phillips et al. 2006; Kishkinev et al. 2021). This conception of magnetic maps proposes that once an individual knows the gradients of key environmental cues, it can extrapolate a homeward direction even if displaced to a distant, unfamiliar area. Thus, the anecdote of a green turtle that returned to its capture site at Ascension Island in the south Atlantic Ocean after being released in the English Channel (Cornelius 1865) might be explained if the turtle sensed the magnetic inclination and intensity and, based on its knowledge of magnetic gradients in the Atlantic, extrapolated that it was north of the island and should swim south, despite being in an unfamiliar location far beyond its natural range. Such feats of navigation, which involve return from unfamiliar locations, are often discussed in the context of ‘true navigation’, a concept formulated nearly 70 years ago (Griffin 1952). Animals are said to be capable of true navigation if, after displacement to a location where they have never been, they can determine their position relative to a goal without relying on familiar surroundings, cues emanating from the destination, or information collected during the outward journey (Griffin 1952; Phillips 1996; Boles and Lohmann 2003). Although the concept of true navigation has shortcomings (e.g., Keeton 1974; Putman 2021), the idea remains useful in discussions of goal navigation in which animals move toward a home area after physical displacement or magnetic displacement to unfamiliar locations.

Several studies have now been done in which turtles, lobsters, birds, and other animals set appropriate courses towards home after magnetic displacements to areas where they had probably never been (e.g., Figs. 3, 5, 6). In this context, a recent study of particular interest involved reed warblers in Austria, which were magnetically displaced to a location well outside of the natural range of the species; the birds responded by orienting in a direction that would return them to Austria (Kishkinev et al, 2021). These findings highlight the accumulating evidence that some animals can, in effect, extrapolate their position along a magnetic gradient, even when in unfamiliar areas.

#### Single-coordinate magnetic maps

As described previously, some seemingly difficult feats of navigation, including natal homing by sea turtles, salmon, and seabirds, can potentially be accomplished without the need for a bicoordinate magnetic map. In principle, many target areas can be reached using only a single magnetic parameter. For example, an animal seeking a coastal goal with a particular intensity or inclination might use the gradients of those parameters along the coastline (e.g., Fig. 7) to assess whether it is north or south of the target location. Emerging findings suggest that different animals in different parts of the world might use different magnetic parameters to recognize coastal locations. For example, analyses suggest that salmon near Vancouver Island use intensity when migrating toward their natal river (Putman et al. 2013); by contrast, Kemp’s ridley turtles (*Lepidochelys kempii*) that nest in northern Mexico, as well as Manx shearwaters that nest along the coast of Great Britain, might use inclination (Putman and Lohmann 2008; Lohmann et al. 2013; Wynn et al. 2020). In each case, the magnetic parameter used may be the one that has been most stable in the natal area during the recent past (Putman and Lohmann 2008; Wynn et al. 2020). Analyses of loggerhead turtle nesting along the Florida coast are consistent with the use of inclination, or intensity, or possibly both (Brothers and Lohmann 2015, 2018).

#### Traveling along an isoline

**Fig. 12:**
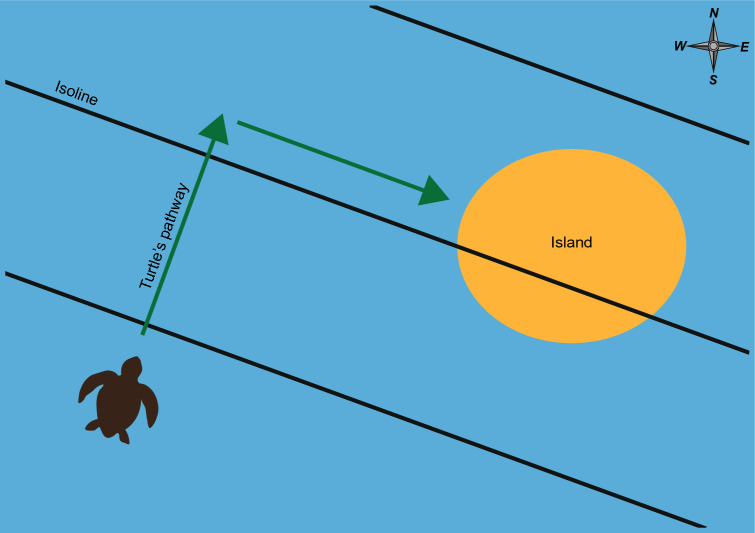
A hypothetical strategy for locating a target area using a single magnetic parameter such as inclination or intensity. Here, a turtle navigates toward an island, knowing either the inclination or intensity of the target. The turtle swims towards the appropriate magnetic isoline but deliberately offsets its route so that it intersects the isoline in a known direction from the island (west of the island in this example). When the turtle encounters the isoline, it turns right and follows the isoline east-southeast until it arrives at the goal. Figure modified from Lohmann et al. (2007)

A single-coordinate navigational strategy can hypothetically be used not only to locate areas along a coast, but also to locate almost any target area (Lohmann et al. 2007). To use this strategy, an animal must know at least one magnetic element (e.g., intensity or inclination) that exists at the goal, so that it can recognize the magnetic isoline on which the destination lies. The animal must then adopt a heading offset to one side or the other of the target, so that when the isoline is intersected, the animal knows which direction to travel along the isoline to reach the goal (Fig. 12). This strategy resembles the ‘parallel sailing’ technique used by mariners at a time when latitude, but not longitude, could be measured reliably (Casey 1993). Rather than attempting to steer directly toward a distant destination, a ship’s navigator deliberately set a course to intersect the appropriate latitude considerably east or west of the target, after which the ship sailed along the latitude in the appropriate direction to reach the target.

The possibility that animals sometimes travel strategically along magnetic isolines, particularly when nearing the endpoint of a migration, has received little attention. Models of sea turtles navigating to islands suggest that a strategy of swimming to a particular isoline, then along it until chemical cues are encountered, would be effective in locating some remote islands (Endres et al. 2016). Similarly, swimming along isolines might lead salmon to spawning grounds under some conditions (Bracis and Anderson 2012). Anecdotal evidence that salmon do indeed swim along isolines came from two tagged chum salmon (*Oncorhynchus keta*), which were found to travel along migratory routes that coincided with isolines of intensity (Azumaya et al. 2016). A strategy of flying to a specific isoline, and exploring along it in both directions for a target area, has also been proposed for birds (Mouritsen 2003). Additional studies are clearly needed.

#### Navigation using magnetic signatures

The gradient hypothesis appears plausible for many magnetic maps that involve goal navigation (e.g., Figs. 3, 5, 6). At the same time, a fundamentally different strategy also potentially exists in which animals might sometimes reach goals using magnetic positional information without relying exclusively—or perhaps at all—on learning the magnetic gradient of an area.

**Fig. 13:**
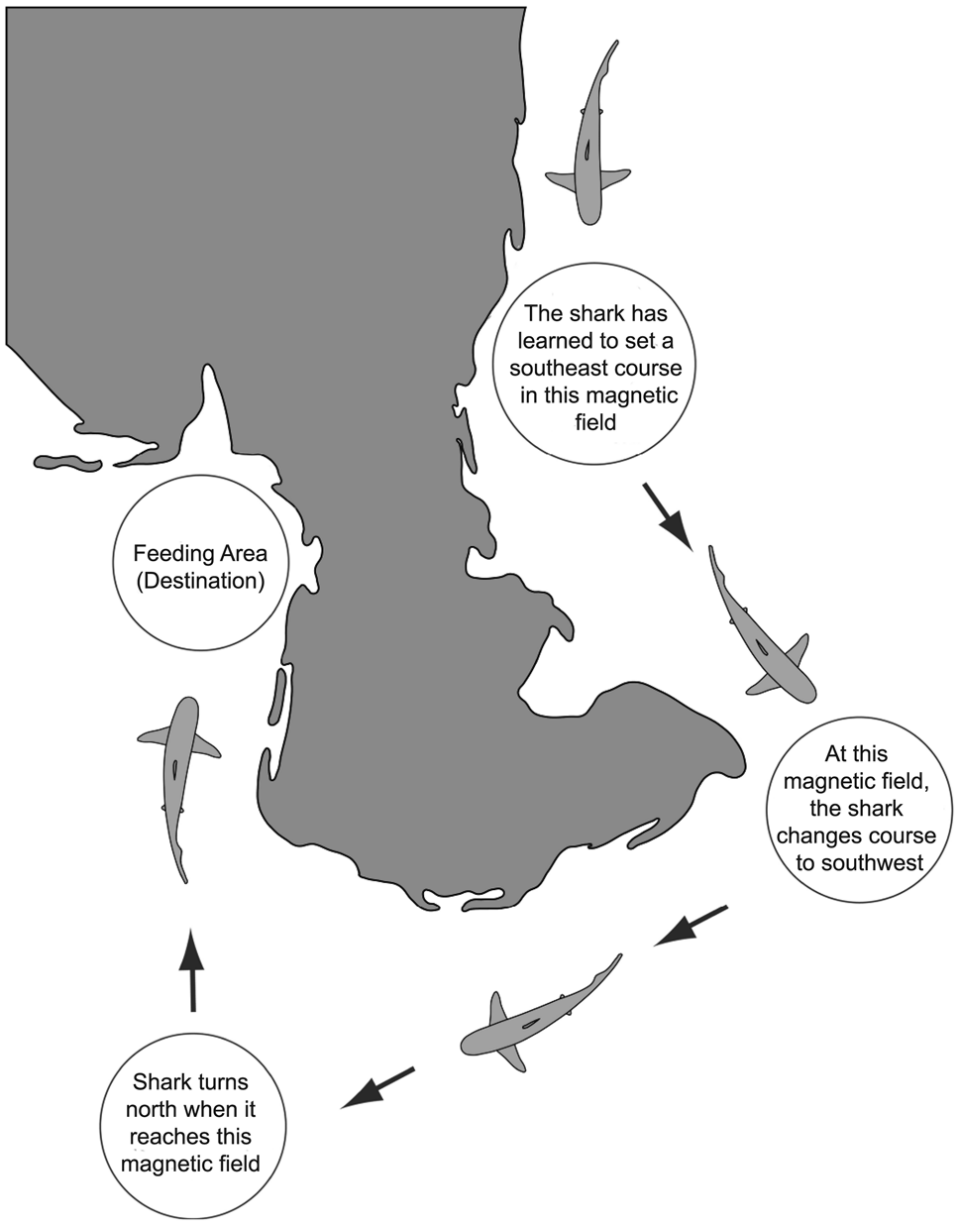
Possible strategy for completing a complex migratory pathway using magnetic signatures. In this hypothetical example, a shark discovers a feeding area on the west side of a peninsula and migrates to it seasonally. As it gains experience, the shark learns the magnetic signatures of several locations along the route, so that the migration is eventually accomplished through a series of steps in which each magnetic location serves as a navigational marker and triggers the appropriate migratory direction for the next segment of the journey. Knowledge of the pattern of regional variation (i.e., the magnetic gradient) is thus not essential. Figure modified from Lohmann et al. (2007)

Through experience, animals might learn to recognize magnetic fields associated with a limited number of important locations along their migratory route, including places where changes in direction are required (Lohmann et al. 2007). In effect, the migration might be carried out as a series of learned steps, with the magnetic field that exists in different locations triggering the appropriate direction for the next leg of the journey (Fig. 13). Simulations of navigation based on magnetic signatures have provided evidence that such a strategy is plausible (Taylor 2018; Taylor and Corbin 2019). An interesting speculation is that the repeated circling of penguins, sea turtles, whales and other ocean migrants observed at some locations (Narazaki et al. 2021) facilitates learning the magnetic field of that area, so that animals can remember the field and use it as a navigational marker on subsequent trips. Of course, learning to recognize magnetic signatures of specific locations and exploiting magnetic gradients are not mutually exclusive; the two strategies might often complement each other.

### Multimodal navigation

An important consideration when discussing magnetic maps is that all animals studied so far use multiple sources of sensory information in orientation and navigation (Lohmann et al. 2008a; Wiltschko and Wiltschko 2015; Wiltschko 2017; Mouritsen 2018). Thus, a magnetic map is not the totality of an animal’s navigational ability, but instead represents just one part of a large and integrated suite of mechanisms. Having a magnetic map does not mean that an animal uses it at all times or under all conditions. Indeed, in many settings, animals can navigate successfully without using magnetic cues at all (Lohmann et al. 2008a).

The extent to which an animal relies on a magnetic map to guide its movement may depend in part on the nature of the environment and the degree to which alternative cues exist. Interestingly, at least two marine animals, loggerhead turtles and pink salmon, appear to rely at least partly on magnetic map information throughout their life cycle, from the first migration that the young undertake to the time when adults return to their natal area to reproduce (Lohmann et al. 2012; Putman et al. 2014a, 2020; Brothers and Lohmann 2015). An intriguing possibility is that magnetoreception is particularly well developed among marine animals, in part because so few other directional and positional cues are available in the open sea to animals that travel well below the surface. By contrast, terrestrial migrants such as birds and insects may have greater access to a wider array of cues including visual landmarks, celestial cues, and windborne odors, among other sources of information.

## Future directions

It is now evident that different animals use magnetic positional information for a variety of purposes, including: (1) staying on track along a migratory pathway; (2) remaining in a favorable oceanic area; (3) adjusting food intake at appropriate points in a migration; (4) moving toward feeding areas, breeding areas, or a home area; and (5) navigating back to an area of origin during natal homing. It is also clear that magnetic maps are phylogenetically widespread, given that animals ranging from lobsters to birds exploit positional information in Earth’s magnetic field.

Despite the explosion of research on magnetic maps, much remains to be learned about their structure, organization, and ontogeny. For example, do general principles exist that are common to all magnetic maps, or has natural selection sculpted a variety of different ways to exploit magnetic map information that vary with species, geographic region, and the navigational task that must be performed? What information is inherited, what is learned, and how do the two types of information interact? How are magnetic maps represented in the brains of animals, and how is the magnetic map sense integrated with other sensory modalities? These and related questions represent an exciting new frontier in behavioral biology.

In less than a generation, the idea that animals use Earth’s magnetic field as a kind of map has gone from a contentious hypothesis to a well-established tenet of animal navigation. The discovery of magnetic maps has ushered in a golden age of research. Solutions to many of the most difficult and seemingly intractable mysteries of animal navigation—including long-distance natal homing, the movement of young marine animals along complex, open-sea migratory pathways, and the ability of animals to home from unfamiliar territory—are now within reach.

## Data Availability

Not applicable.
